# Quantitative Receptor-Based Imaging of Tumor Proliferation with the Sigma-2 Ligand [^18^F]ISO-1

**DOI:** 10.1371/journal.pone.0074188

**Published:** 2013-09-20

**Authors:** Kooresh I. Shoghi, Jinbin Xu, Yi Su, June He, Douglas Rowland, Ying Yan, Joel R. Garbow, Zhude Tu, Lynne A. Jones, Ryuji Higashikubo, Kenneth T. Wheeler, Ronald A. Lubet, Robert H. Mach, Ming You

**Affiliations:** 1 Department of Radiology, Washington University School of Medicine, St. Louis, Missouri, United States of America; 2 Department of Biomedical Engineering, Washington University School of Medicine, St. Louis, Missouri, United States of America; 3 Department of Surgery, Washington University School of Medicine, St. Louis, Missouri, United States of America; 4 Department of Cell Biology and Physiology, Washington University School of Medicine, St. Louis, Missouri, United States of America; 5 Department of Biochemistry and Molecular Biophysics, Washington University School of Medicine, St. Louis, Missouri, United States of America; 6 Division of Biology and Biomedical Sciences, Washington University School of Medicine, St. Louis, Missouri, United States of America; 7 Center for Molecular and Genomic Imaging, University of California Davis, Davis, California, United States of America; 8 Department of Radiology, Wake Forest University Health Science Center, Winston-Salem, North Carolina, United States of America; 9 National Institutes of Health, Bethesda, Maryland, United States of America; 10 Cancer Center and Department of Pharmacology and Toxicology, Medical College of Wisconsin, Milwaukee, Wisconsin, United States of America; Vanderbilt University, United States of America

## Abstract

The sigma-2 receptor is expressed in higher density in proliferating (P) tumor cells versus quiescent (Q) tumor cells, thus providing an attractive target for imaging the proliferative status (i.e., P:Q ratio) of solid tumors. Here we evaluate the utility of the sigma-2 receptor ligand 2-(2-[^18^F]fluoroethoxy)-*N*-(4-(3,4-dihydro-6,7-dimethoxyisoquinolin-2(1*H*)-yl)butyl)-5-methyl-benzamide, [^18^F]ISO-1, in two different rodent models of breast cancer. In the first study, small animal Positron Emission Tomography (PET) imaging studies were conducted with [^18^F]ISO-1 and ^18^FDG in xenografts of mouse mammary tumor 66 and tracer uptake was correlated with the *in vivo* P:Q ratio determined by flow cytometric measures of BrdU-labeled tumor cells. The second model utilized a chemically-induced (*N*-methyl-*N*-nitrosourea [MNU]) model of rat mammary carcinoma to correlate measures of [^18^F]ISO-1 and FDG uptake with MR-based volumetric measures of tumor growth. In addition, [^18^F]ISO-1 and FDG were used to assess the response of MNU-induced tumors to bexarotene and Vorozole therapy. In the mouse mammary 66 tumors, a strong linear correlation was observed between the [^18^F]ISO-1 tumor: background ratio and the proliferative status (P:Q ratio) of the tumor (R = 0.87). Similarly, measures of [^18^F]ISO-1 uptake in MNU-induced tumors significantly correlated (R = 0.68, P<0.003) with changes in tumor volume between consecutive MR imaging sessions. Our data suggest that PET studies of [^18^F]ISO-1 provide a measure of both the proliferative status and tumor growth rate, which would be valuable in designing an appropriate treatment strategy.

## Introduction

In the clinical management of cancer, major research efforts are devoted to the optimization of chemo- and radio-therapies. To that end, Positron Emission Tomography (PET) is widely used in diagnosis and detection of cancer as well as in characterizing therapeutic efficacy. One of the most commonly used radiopharmaceuticals in oncological imaging is ^18^F-labeled 2-fluoro-2-deoxy-d-glucose (FDG). FDG is an analog of glucose with the oxygen at the 2′ position replaced by fluorine and is taken up in tissue via the same transport mechanism as glucose. Subsequent to phosphorylation by the enzyme hexokinase to yield FDG-6-phosphate, it is trapped in tumors, thus providing a non-invasive measure of glucose utilization. PET measures of glucose metabolism have been used in the diagnosis and staging of cancer, as well as in assessing efficacy of therapeutic intervention [1], [2]. However, it has been well established that FDG measures of tumor metabolism do not correlate with tumor cell proliferation [3]. To that end, radiolabeled nucleosides, including [^18^F]fluorine-labeled thymidine ([^18^F]FLT) which measures the salvage pathway of DNA synthesis, have been developed as PET radiotracers for measuring tumor proliferation rate [4].

Much like FDG, [^18^F]FLT is trapped within the cell as the corresponding [^18^F]FLT-6-phosphate. This is attributed to the action of the enzyme thymidine kinase-1 (TK1) which phosphorylates the 6′-hydroxyl group of [^18^F]FLT. TK1 is catalytically active only during the S phase; therefore, [^18^F]FLT uptake provides a measure of the S-phase fraction of a tumor. PET is a pulse labeling technique; i.e., the radiotracer is administered intravenously and the imaging study is conducted within a narrow period of time (30–120 min post-injection). Because tumors exhibit asynchronous growth and contain cells in every phase of the cell-cycle, G1, S, G2 and M phases, a pulse label measurement of the S-phase fraction of a tumor with [^18^F]FLT is expected to underestimate the total number of proliferating cells. Furthermore, most solid tumors contain a population of quiescent tumor cells (i.e., G0 cells) which exit the cell cycle due to nutrient deprivation. Taken together, solid tumors are intrinsically heterogeneous and contain populations of tumor cells that are either proliferating (P) or quiescent (Q). Since radiotherapy and most chemotherapeutic agents target proliferating tumor cells more effectively than quiescent cells, knowledge of the proliferative status (i.e., P:Q ratio) of a tumor can be used to design appropriate radio- or chemo- therapy treatment strategies [5]. For example, hyperfractionated radiation therapy or cell-cycle-specific therapeutic agents can be used in tumors with a high proliferative status [6], [7]. Conversely, in tumors with a low proliferative status, non-cell-cycle-specific agents can be used [6]. Finally, the proliferative status of a tumor can be used to identify patients who will benefit from therapies targeting proteins expressed in cycling cells but absent in quiescent tumor cells [8], [9].

Historically, the proliferative status of a solid tumor has been measured by counting the number of Ki-67 positive cells in a biopsy specimen. Ki-67 is a nuclear protein which is expressed in all phases of the cell cycle of proliferating tumor cells except in quiescent tumor cells [10]. Therefore, a PET radiotracer which could image Ki-67 would provide a method for measuring the proliferative status of solid tumors. Unfortunately, there are currently no small molecules that bind with high affinity to Ki-67 reported in the literature which could serve as lead compounds for PET radiotracer development. Consequently, attempts to develop a PET radiotracer for imaging the proliferative status of a tumor have relied on the identification of other biomarkers which behave in a manner analogous to Ki-67. One possible candidate protein is the sigma-2 receptor which has been shown to be overexpressed in a variety of tumors [11]–[13]. In previous studies using the mouse mammary tumor 66 cell line, the density of sigma-2 receptors was found to be 10-fold higher in proliferating 66 versus quiescent 66 cells in vitro [14]. This observation was later confirmed in solid tumor xenografts [15], suggesting that in vivo measures of the sigma-2 receptor status may reflect the proliferative status of a solid tumor. To that end, both ^11^C- and ^18^F-radiolabeled sigma-2 receptor ligands have been developed [16], [17] and validated in a variety of tumor models [15], [17], [18]. More recently, we have published the first-in-human study using [^18^F]ISO-1 and reported significant correlation between image measures of [^18^F]ISO-1 and Ki-67 as a marker for tumor proliferation [19].

In this work, the sigma-2 receptor ligand 2-(2-[^18^F]fluoroethoxy)-*N*-(4-(3,4-dihydro-6,7-dimethoxyisoquinolin-2(1*H*)-yl)butyl)-5-methylbenzamide, [^18^F]ISO-1 [16], was used to assess two clinically-relevant properties of breast tumors: a) measurement of the proliferative status of the tumor; and, b) prediction of the volumetric change associated with tumor growth. The goal of the first study was to correlate measures of [^18^F]ISO-1 uptake with measures of tumor proliferative status as determined by BrdU-labeling and ex vivo flow cytometry analysis performed on mouse 66 tumors. The second study employed a chemically-induced (i.e., *N*-methyl-*N*-nitrosourea [MNU]) rat model of mammary carcinoma [20] to correlate [^18^F]ISO-1 uptake with measures of tumor growth rate determined by MRI-derived measures of tumor volume. MNU-induced tumors are hormonally responsive and display many of the histopathological characteristics of human breast cancer [21]. Unlike clonogenic cancer models, individual *in situ* MNU-induced tumors have a highly variable growth rate [22]. Therefore, multi-modality imaging was performed using small animal MR and PET to correlate FDG and [^18^F]ISO-1 image-derived PET outcome measures as a means of predicting future changes in tumor volume. The MNU-model of breast cancer has also been employed in the development and preclinical evaluation of a number of chemotherapeutics. Therefore, a final goal of this study was to assess the ability of FDG and [^18^F]ISO-1 to monitor the response to chemotherapy. The therapeutic agents that were evaluated in this work were bexarotene, which belongs to a class of chemical compounds targeting the retinoid × receptors (RXR), and Vorozole, a competitive inhibitor of the aromatase enzyme.

## Materials and Methods

All animal studies were approved by an independent Washington University Animal Study Committee (Protocols #20090004, 20100107).

### Synthesis of Radiolabeled Ligands

The tritiated sigma-2 receptor ligand [^3^H]RHM-1 was synthesized by American Radiolabeled Chemicals, Inc. (St. Louis, MO) via *O*-alkylation of the corresponding phenol precursor [17]; chemical purity was greater than 99% and the specific activity of the radiolabeled ligand was 80 Ci/mmol. FDG is routinely synthesized at the Washington University Cyclotron facility. The synthesis of [^18^F]ISO-1 has been described previously [15], [17], [18].

### Validation of In-Vivo Measures of Tumor Proliferation using Mammary 66 Tumors

#### Cell culture and implantation of 66 mammary tumors

The mouse mammary 66 cells were cultured as previously described [15]. Approximately 1.5×10^6^ cells/100 µL were injected subcutaneously in the axillary regions of adult (20–25 g) female nude mice to produce the bilateral tumors. Two to three weeks after implantation, the P-cells in each tumor were identified by labeling them with BrdU (Calbiochem-Novabiochem Corp., La Jolla, CA, USA). Mice were injected with 100 mg/kg of BrdU intraperitoneally (i.p.) every 8 h over a 48-h period (i.e. for at least 2 cell cycle times). At the time of labeling, the tumors ranged in size from 0.2 g to 1.0 g.

#### Imaging protocol and analysis

Prior to imaging mice were anesthetized with 2% 2.5% isoflurane by inhalation via an induction chamber. Anesthesia was maintained throughout the imaging session by delivering 1%–1.5% isoflurane via a custom-designed nose cone on a custom designed mice holder. The mammary 66 tumor-bearing mice were secured side-by side and placed inside the field of view (FOV) of the small animal imaging PET scanner. After a transmission scan was performed, a 10-minute static acquisition study was obtained approximately sixty (60) minutes following injection of [^18^F]ISO-1 or [^18^F]FDG (∼200 µCi). Images were reconstructed using Filtered Back Projection (FBP). Regions of interest (ROI) were manually drawn on the tumors and appropriate reference regions delineated with the software Acquisition Sinogram Image PROcessing using IDL’s Virtual MachineTM (ASIPro VMTM) to obtain the radioactivity uptake (nCi/mL) in each tumor and the surrounding background tissue. Tumor to background radioactivity uptake ratios are further compared with P:Q ratios described in the next sections.

#### BrdU labeling and flow cytometry analysis of the P:Q ratio

Animals were euthanized and tumors were excised, minced and dissociated with an enzyme cocktail consisting of 0.04% collagenase, 0.04% pronase (∼2500 PUK/100 mL) and 0.05% DNAase I in Waymouth’s medium without serum. After incubating for 30–45 min at 37°C with continuous stirring, the material was filtered through an 80-mesh screen. The filtrate was centrifuged at 225×G at 4°C for 5 minutes, and the pellet resuspended in Waymouth’s medium with 10% serum (to halt the enzyme action) and held on ice while an aliquot was counted. Single cell suspensions were then centrifuged again, resuspended in phosphate-buffered saline (PBS) and fixed in 70% ethanol to obtain a final concentration of 1–2×10^7^ cells/mL.

For the flow cytometry analysis, 1.5×10^6^ cells were first incubated for 20 min at 37°C with 0.2 mg/mL of pepsin in 2 N HCl–PBS, washed twice in PBS containing 0.5% fetal bovine serum (FBS), and then incubated for 45 min with a mouse anti-BrdU antibody conjugated to fluorescein isothiocyanate (Boehringer Mannheim, Indianapolis, IN, USA). The cells were then washed in 1 mL of PBS containing 0.5% FBS and 0.5% Tween-20, incubated for 30 min in RNAase (1 mg/mL) and stained with propidium iodide (10 mg/mL). All flow cytometry was performed using a Fluorescence-Activated Cell Sorting (FACS) instrument (BD Biosciences, San Jose, CA, USA) equipped with an air-cooled argon laser using an excitation wavelength of 488 nm. The gating parameters in each experiment were set to insure that less than 2% of the unlabeled cells had a fluorescence signal equivalent to that of the weakest BrdU labeled cells in the tumor. Proliferative (P) to quiescent (Q) cells ratio, P:Q ratio, is defined by BrdU labeled positive cell fraction divided by negative cell fraction.

#### Correlation between [^18^F]ISO-1, FDG PET measures and P:Q ratio

PET outcome measures were compared with the tumor proliferative status, BrdU labeled P:Q ratio. Radiotracer tracer uptake in the whole tumor to the surrounding background tissue ratio was plotted against the P:Q ratio and regression analysis were performed using KaleidaGraph (Synergy Software, Reading, PA, USA).

### MNU-Induced Mammary Carcinoma Tumor Proliferation and Response to Therapy

#### Animal study design

At 50–60 days of age, female Sprague-Dawley rats were injected i.v. with 50 mg/kg MNU. Rats were selected for the study when at least one palpable tumor was apparent. The study employed 30 untreated MNU rats, 6 MNU rats treated with bexarotene, and 6 MNU rats treated with Vorozole. Bexarotene (220 mg/kg in the diet) and Vorozole (1.25 mg/kg body weight by gavage) were provided for 8-weeks following a baseline imaging session. Following the 8^th^ week imaging session, treatment was removed and rats were fed AIN-76 diet. Untreated rats were fed AIN-76 diet throughout the time course of the study. Each rat was imaged with FDG over a 10-week period at 2-week intervals to assess the metabolic state of tumors, MRI to monitor tumor volume, and [^18^F]ISO-1 to assess sigma-2 receptor status. A subset of untreated rats was used in determination of sigma-2 receptor density described below. Due to logistical constraints, MRI and PET images were typically acquired within ±1 day.

#### In-vitro determination of sigma-2 receptor density

Sigma-2 receptor binding studies using tumor homogenate were conducted as previously described in other tissues [23]. Tumor membrane homogenate was diluted with 50 mM Tris-HCl buffer, pH 8.0 and incubated for 60 min with [^3^H]RHM-1 in a total volume of 150 µL at 25°C in 96 well polypropylene plates. The concentrations of the radioligand ranged from 0.1–18 nM. After incubation the reactions were terminated by the addition of 150 µL cold wash buffer, and the samples harvested and filtered rapidly to a 96 well fiber glass filter plate. Each filter was washed for a total of three washes. A liquid scintillation counter was used to quantitate the bound radioactivity. Nonspecific binding was determined from samples which contained 10 µM haloperidol. The equilibrium dissociation constant (*K_d_*) and maximum number of binding sites (B_max_) were determined by a linear regression analysis of the transformed data using the method of Scatchard [24]. Data from saturation radioligand binding studies was transformed to determine the Hill coefficient, nH, defined as
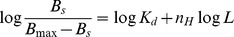



B_s_ is the amount of the radioligand bound specifically; L is the concentration of radioligand. nH, Hill slope, was determined from Hill plot of
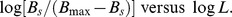



#### Pre-clinical MR imaging protocol

High-resolution MRI was used to monitor tumor volume and morphology in all rats with mammary tumors. MR images were collected in an Oxford Instruments (Oxford, UK) 4.7 Tesla magnet (33 cm, clear bore) equipped with 16-cm, inner-diameter, actively shielded Oxford gradient coils (maximum gradient 18 G/cm, 200 µsec rise time) and high-power IEC gradient amplifiers (International Electric Co, Helsinki, Finland). The magnet/gradients are interfaced with a Varian (Palo Alto, CA) INOVA console and data were collected using a Stark Contrast (Erlanger, Germany) 5-cm birdcage rf coil. Prior to the imaging experiments, rats were anesthetized with isoflurane and were maintained on isoflurane/O_2_ (1–1.5% v/v) throughout the experiments. Multi-slice (20–25 slices), T_2_-weighted, transaxial spin-echo images (T_r_ = 1.0–1.7 s; T_e_ = 40 ms, FOV = 5 cm×5 cm; slice thickness = 1 mm) were collected for each rat. Tumor MR images appeared as distinct, hyperintense regions, whose borders with normal tissue was easily and unambiguously delineated. Tumor growth was measured by manually segmenting individual tumors in each image and calculating volumes using either Varian’s Image Browser software or the public domain program ImageJ (http://rsb.info.nih.gov/ij).

#### Pre-clinical PET imaging protocol

Small-animal PET was performed on either the microPET® Focus-120 [25] or Focus-220 [26] (Siemens Inc., Knoxville, TN). Both scanners were cross-calibrated to a common source.

*Dynamic PET Imaging with [^18^F]ISO-1*. Dynamic imaging studies were performed to characterize the pharmacokinetics of [^18^F]ISO-1. Prior to imaging rats were anesthetized with 2% 2.5% isoflurane by inhalation via an induction chamber. Anesthesia was maintained throughout the imaging session by delivering 1%–1.5% isoflurane via a custom-designed nose cone and rat holder. MNU-treated rats were secured and were placed inside the field of view (FOV) of the small-animal imaging PET scanner. A bolus injection of [^18^F]ISO-1 was administered via the tail vein approximately 5 seconds after the start of the PET scan and a dynamic acquisition study was conducted for 120 min. Images were reconstructed using Filtered Back Projection (FBP). ROIs were drawn on PET images to characterize the kinetic time-course of [^18^F]ISO-1 in tumor, muscle, and the left-ventricle (LV) to characterize its time-course in blood. Muscle ROIs were drawn by placing a similar ROI as the tumor ROI on the contralateral side of the animal. Tumor-to-blood and tumor-to-muscle ratios were at steady state at 60 min suggesting that static imaging 60 min post-injection of [^18^F]ISO-1 would capture tumor uptake of the ligand.

*Static PET Imaging with FDG and [^18^F]ISO-1*. Sixty (60) minutes following injection of FDG (0.6–0.8 mCi) or [^18^F]ISO-1 (0.6–0.8 mCi), rats were secured in a custom-designed acrylic restraining device and placed inside the field of view (FOV) of the small animal imaging PET scanner, as described above. A 10-minute static acquisition ensued. Images were reconstructed using Filtered Back Projection (FBP).

#### Multi-modality image analysis and processing

Individual tumors in MRI and PET images were carefully matched by an experienced technician to localize tumors. The same technician processed all images to minimize inter-operator variability. MRI images were used as landmarks to identify tumors on PET images. Tumor FISO and FDG uptake were consistently higher than surrounding tissue; hence, easily distinguishable. Care was taken to draw ROIs on the entire tumor boundary as visualized by a experienced technician. Volumes of interest (VOIs) masks were drawn on PET images using the program Analyze (Biomedical Imaging Resource, Mayo Clinic). Masked images were subsequently imported into Matlab (Mathworks, Inc.) where the activity concentration in each voxel was standardized by the activity injected and the animal weight (SUV = (activity concentration in voxel)*(animal weight)/(activity injected)). To facilitate data mining, a database was constructed for each tumor that included the PET image mask, MRI-derived volume, PET-derived volume, activity injected, animal weight, and PET signal distribution within the tumor for each radiopharmaceutical as a function of time. The dataset was used to tabulate PET and MRI outcome measures, which were used to assess the predictive capacity of FDG and [^18^F]ISO-1.

#### Correlation between FDG and [^18^F]ISO-1 PET outcome measures and tumor growth pattern

PET outcome measures were compared with changes in MRI-derived volume outcome measures. Specifically, all but PET volume outcome measures were correlated against absolute and relative percent change in consecutive volume measurements (i.e., consecutive pairs: 0–2 weeks, 2–4 weeks, 4–6 weeks, 6–8 weeks, and 8–10 weeks). Let *j* denote the imaging session at week *j* (*j* = 0, 2, 4, 6, 8, 10), PET outcome measures at week *j* were correlated to absolute or percent change in volume measure between week (*j+2)* and *j*. Absolute change in tumor volume is defined as the difference in MRI-derived volume between week (*j+2*) and *j*, i.e., [*V*(*j+2)-V*(*j*)] where *V* denotes volume. Relative percent change in volume is defined as absolute change in volume relative to week *j,* i.e.,100* [*V(j+2)-V*(*j*)]/V(*j*). In this data mining approach, the effects of necrosis was accounted for by taking into account the distribution of tracer uptake in the whole tumor. The strength of the correlation between measures was determined by regression analysis performed using the statistical package SPSS.

#### Assessing efficacy of therapy

Tumor response to bexarotene and Vorozole was characterized in terms of MR-derived tumor load, metabolic state (FDG), and sigma-2 receptor status ([^18^F]ISO-1). Both MR- and PET-derived measures were normalized to baseline for ease of analysis. Aside from tumor’s response to therapy, we also considered the short-term efficacy 2-weeks following treatment and effects of withdrawing treatment, both characterized by tumor volume and changes in PET outcomes measures relative to baseline. In reporting PET measurements, treated tumors were classified by the nature of their response to therapy: tumors that exhibited reduced volume by the end of the study (or last time-point) are denoted by a solid line; otherwise, tumors which did not respond to therapy were denoted by a dashed line.

### Statistical Analysis

Correlation analyses were performed using either KaleidaGraph (Synergy Software, Reading, PA, USA) or SPSS (IBM). In assessing significance of correlation a P-value P<0.05 was considered significant.

## Results

The goal of the current study was to assess the capacity of FDG and the sigma-2 receptor ligand, [^18^F]ISO-1, to measure two different clinically-relevant properties of solid tumors, proliferative status (P:Q ratio) and tumor growth rate. FDG was chosen as the reference ligand for comparison with [^18^F]ISO-1 since it is currently the “gold standard” for PET oncological imaging studies. Mouse mammary 66 tumors were used to correlate *in-vivo* measures of radiotracer uptake to *ex vivo* measures of the P:Q ratio since this cell line has been shown to produce solid tumors having a highly variable P:Q ratio [15], [27], [28]. MNU-induced tumors were also used as a more clinically relevant model of mammary carcinoma, both in terms of etiology and progression. MNU tumors were imaged for 10 weeks at 2-week intervals with MRI to characterize tumor volume, FDG to measure tumor’s metabolic state, and [^18^F]ISO-1 as a potential predictive imaging marker for tumor proliferation. PET-derived outcome measures were subsequently correlated to changes in tumor volume. Finally, the response of MNU-induced tumors to two different chemotherapies used to treat breast cancer was assessed with both FDG and [^18^F]ISO-1.

### Correlation of FDG and [^18^F]ISO-1 Uptake with P:Q ratio

**Figure 1:**
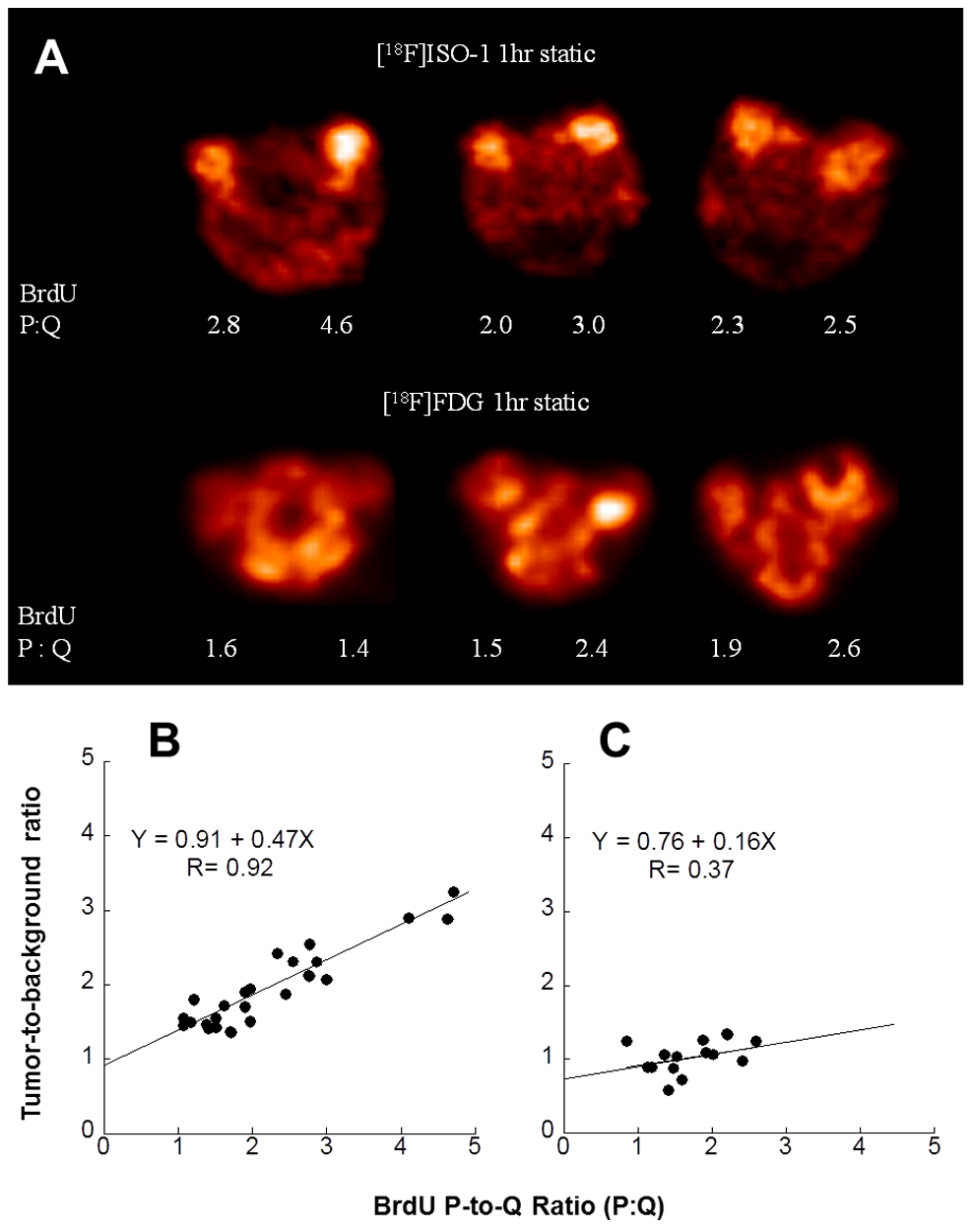
Validation of in-vivo measures of tumor proliferation using mammary 66 tumors. (**A**) Sum microPET images of sigma-2 ligand [^18^F]ISO-1 (top panel) and [^18^F]FDG (bottom panel) in 66 mammary tumors with varying P:Q ratios. Correlation between [^18^F]ISO-1**,** [^18^F]FDG PET measures and P:Q ratio. Radiotracer tracer uptake tumor to background ratio was plotted against the P:Q ratio and linear regression analysis were performed. (**B**) [^18^F]ISO-1 tumor to background ratio linearly correlates with P:Q ratio with a correlation coefficient R = 0.92 and steep slope of 0.47; (**C**) [^18^F]FDG tumor to background ratio slightly correlates with P:Q ratio, R = 0.37, with a slope of 0.16.

Tumors from the 66 cell line having a wide range of P:Q ratios were imaged with either [^18^F]ISO-1 or FDG. As shown in Figure 1A, [^18^F]ISO-1 yielded good contrast, with the tumor to background ratio comparable to the metabolic radiotracer FDG. A strong linear correlation of R = 0.87 was observed between the tumor: background ratio and P:Q ratio for [^18^F]ISO-1 (Figure 1B), whereas the correlation of FDG was poor, R = 0.37 (Figure 1C). This study indicates that PET imaging targeting sigma-2 receptor can potentially predict the proliferative status of a solid tumor in vivo.

### [^18^F]ISO-1 Kinetics and Sigma-2 Receptor Density in MNU-induced Tumor

**Figure 2:**
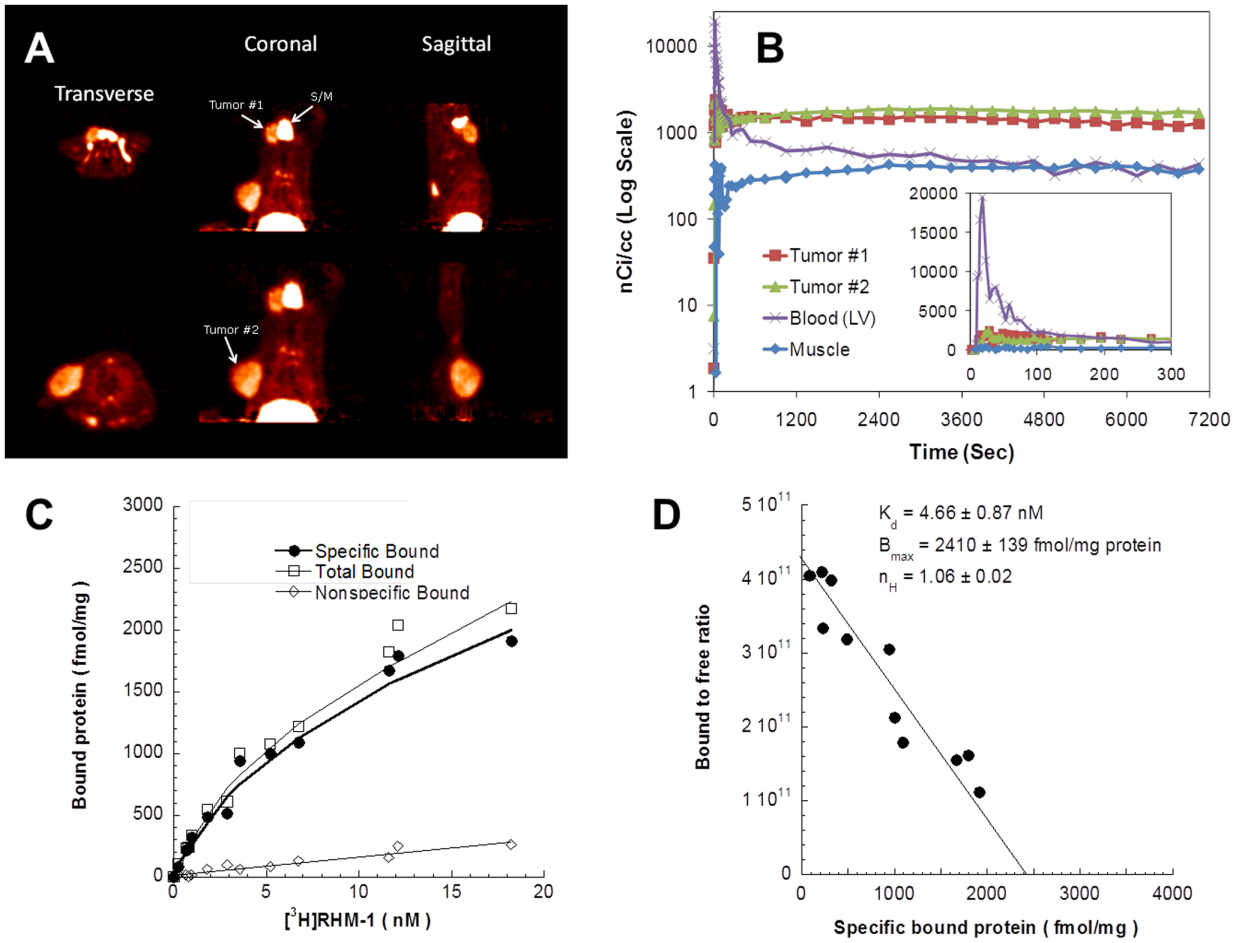
Characterization of the pharmacokinetics of [^18^F]ISO-1 and in-vitro determination of Sigma-2 Receptor Density. (**A**) A 2-hour sum image depicting two MNU-induced tumors and the submandibular (S/M). The liver is evident in the coronal slices. (**B**) Time activity curves of the two tumors, muscle, and the left-ventricular blood pool. Inset figure depicts kinetics at initial 5 min. (**C**) Representative saturation binding experiments which show the total bound, non-specific bound and specific bound. (**D**) Representative Scatchard plots which were used to determine *K_d_*, *B_max_* and *n*_H_ values.

Representative images of [^18^F]ISO-1 in MNU-induced tumors are depicted in Figure 2A. MNU-induced tumors exhibit significant uptake of [^18^F]ISO-1 with minimal background uptake. Additionally, there is high uptake of [^18^F]ISO-1 in the submandibular (S/M) gland, a region known to exhibit high proliferative activity [29] and demonstrated blocking of non-selective sigma 1/2 PET ligands [30]. The kinetics of [^18^F]ISO-1 in MNU-induced tumors is depicted in Figure 2B. Tumor:blood and tumor:muscle ratios achieved steady state after 60 min suggesting that static imaging 60 min post-injection of [^18^F]ISO-1 would measure tumor uptake. To characterize the receptor density (B_max_) of [^18^F]ISO-1, direct saturation binding studies were carried out using [^3^H]RHM-1 with membrane homogenate from a MNU-induced rat mammary tumor. The saturation curve and Scatchard plots are shown in Figure 2C and Figure 2D, respectively. The *K*_d_ and *B*_max_ values of the receptor-radioligand binding of [^3^H]RHM-1 were 4.66 nM and 2410 fmol/mg protein, respectively. The mean of the Hill coefficient (*n*_H_ values) was found to be near unity, which is consistent with one-site fit. The high sigma-2 receptor density and low non-specific binding further supports the use of the MNU-induced mammary tumor model for the in vivo evaluation of [^18^F]ISO-1.

### Time Course of MNU-Induced Tumor Growth

**Figure 3:**
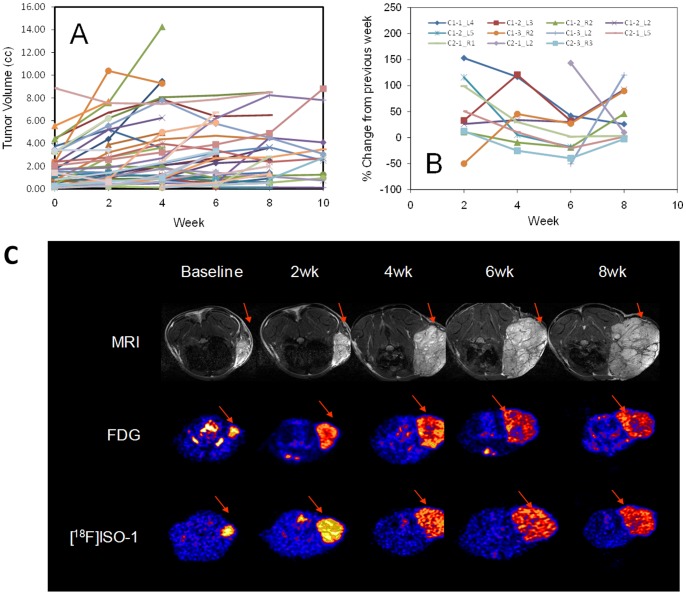
MR Tumor volume measured at baseline and at 2-week intervals. (A) MR-derived tumor volume (B) Percent change in tumor volume between consecutive imaging sessions for a select group of tumors. In analyzing the data, we exploited on the lack of consistency in tumor increase and correlated changes in tumor volume to baseline measures of normalized [^18^F]ISO-1 uptake with the underlying notion that change in tumor volume is predicated on the proliferative status of the tumor. (C) Representative time course imaging of MNU-induced tumors at baseline, 2 weeks, 4 weeks, 6 weeks, and 8 weeks with MRI, FDG and [^18^F]ISO-1. Note: time-course images are on differing color scale for each time point.

Figure 3 displays the time-course of MNU-induced tumor progression, both in terms of tumor growth (Figure 3A) and percent change in tumor volume between consecutive imaging sessions for select tumors (Figure 3B). There was a large variability in tumor growth patterns (Figure 3B). In general, large tumors are characterized by decreasing growth rate although the pattern is not consistent. Moreover, Figure 3B suggests that the tumor’s doubling time is not constant as it may be in fact be dictated by its environment and other exogenous factors.

**Figure 4:**
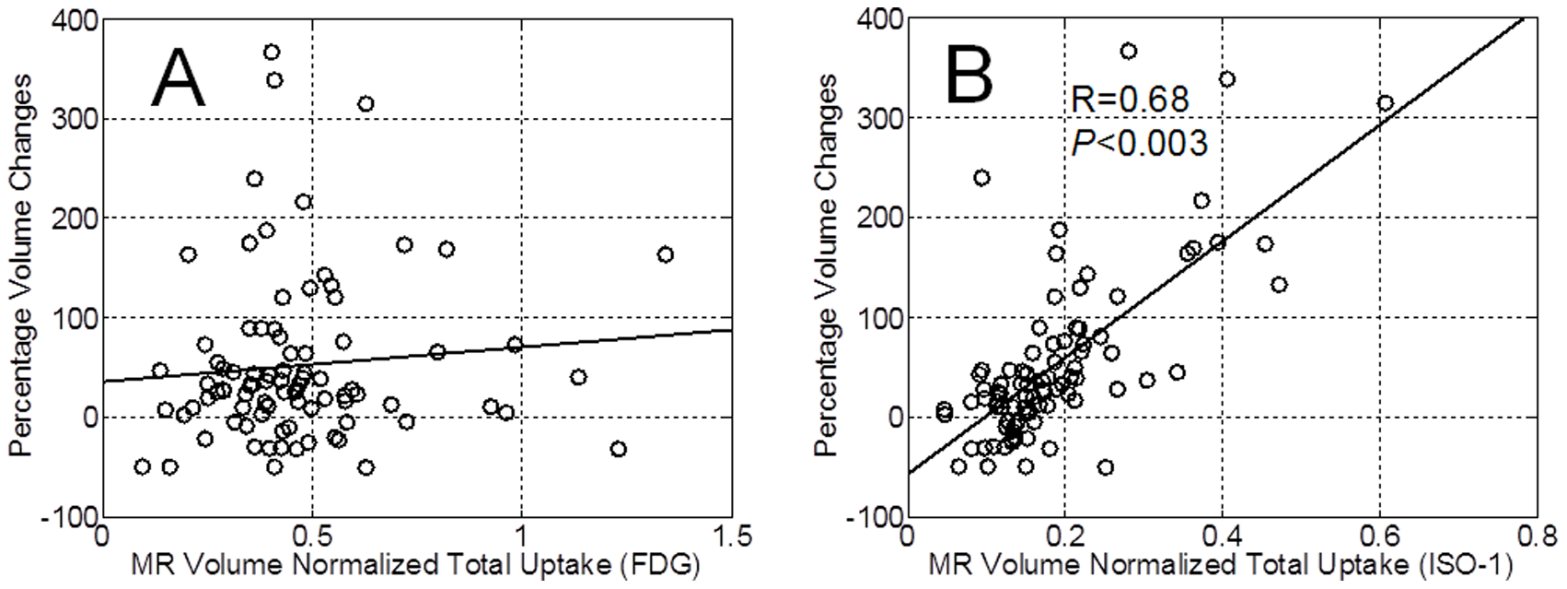
Correlation of PET measures of tumor proliferation to subsequent changes in tumor volume. (**A**) Correlation between normalized FDG total uptake and percent change in volume between consecutive imaging sessions in untreated MNU rats. (**B**) Correlation between normalized [^18^F]ISO-1 total uptake and percent change in volume between consecutive imaging sessions in untreated MNU rats. The correlation coefficient is denoted by R = 0.68 significant at P<0.003. MRI and PET images were typically acquired within a ±1 day, which could explain some of the variability along the fitted line in Figure 7B.

Representative time-course multi-modality imaging with MRI, FDG, and [^18^F]ISO-1 is depicted in Figure 3C. Using the time-course data, the tumor percent change in volume between consecutive imaging sessions was determined. The normalized FDG and [^18^F]ISO-1 SUV uptake at a given imaging time point were subsequently correlated to the change in tumor volume by the time of the next imaging session (2 weeks). While there was no significant correlation between FDG SUV total uptake and percent change in volume (Figure 4A), there is a significant (P<0.003) correlation (R = 0.68) between [^18^F]ISO-1 total uptake SUV and percent change in volume between consecutive imaging session (Figure 4B).

### Tumor Response to Treatment

#### Tumor load measurements

**Figure 5:**
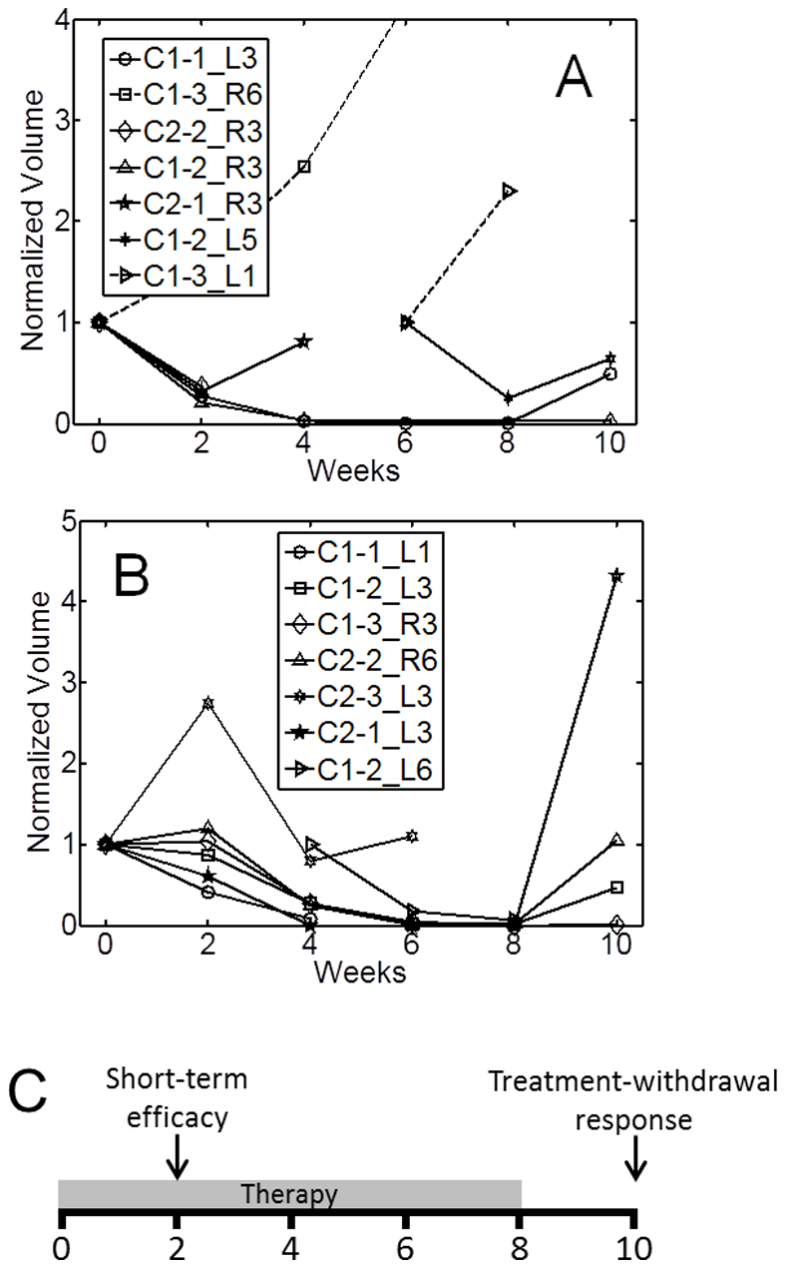
Normalized tumor volume in bexarotene- treated MNU rats (A) and Vorozole (B). The legend in each plot denotes tumor ID which matches tumor IDs of Figure 6, where applicable. Treatment was provided as described in the methods section for 8 weeks. Following the imaging session at week 8, treatment was withdrawn. (**C**) Time course of treatment is segmented into the short term efficacy (week 0–2) and tumor response to treatment withdrawal (weeks 8–10).

The therapeutic effects of bexarotene and Vorozole on tumor load are depicted in Figure 5A and Figure 5B, respectively. Bexarotene appeared to have the stronger short-term efficacy at 2-weeks with a reduction in tumor load, on average, by as much as 60% compared with Vorozole’s 20% reduction in tumor load. However, six weeks following the initiation of treatment both agents induced strikingly reduced the volume of most tumors. Removal of therapeutic agent from the diet resulted in resurgence of tumors. We observed more rapid tumor resurgence in rats treated with Vorozole compared with bexarotene, suggesting a residual effect of bexarotene. In most cases, MR imaging detected more tumors than PET owing to the higher spatial resolution and larger field of view of the MR scanner.

#### Bexarotene-treated rats

**Figure 6:**
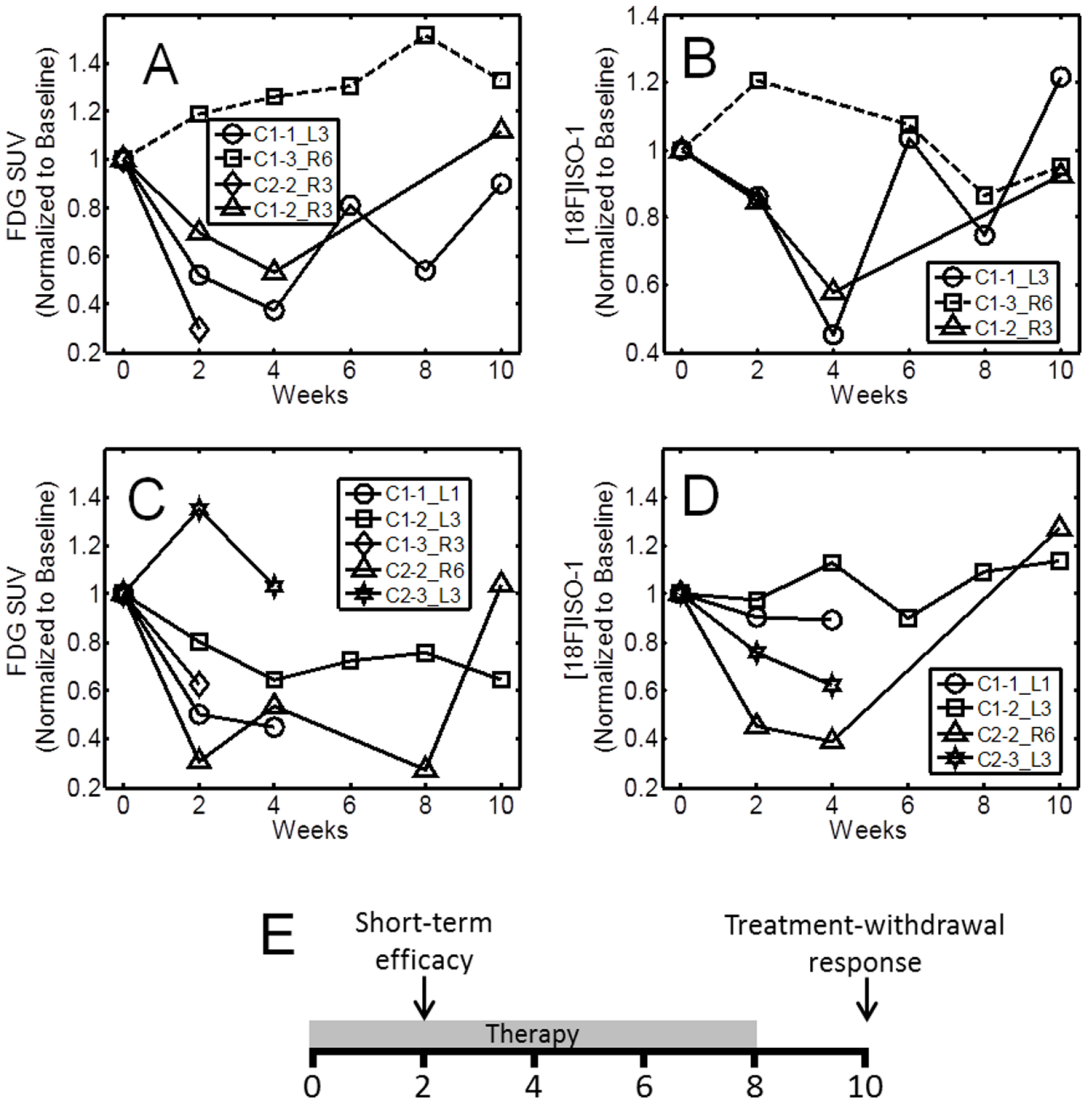
Time-course of FDG and [^18^F]ISO-1 mean SUV normalized to baseline for bexarotene- and Vorozile-treated MNU rats. Top row: (**A**) FDG mean SUV and (**B**) [^18^F]ISO-1 mean SUV for bexarotene-treated rats. Bottom row: (**C**) FDG mean SUV and (**D**) [^18^F]ISO-1 mean SUV for Vorozole-treated rats. Treatment was provided for 8 weeks. Following the imaging session at week 8, treatment was withdrawn. (**E**) Time course of treatment is segmented into the short term efficacy (week 0–2) and tumor response to treatment withdrawal (weeks 8–10).

**Figure 7:**
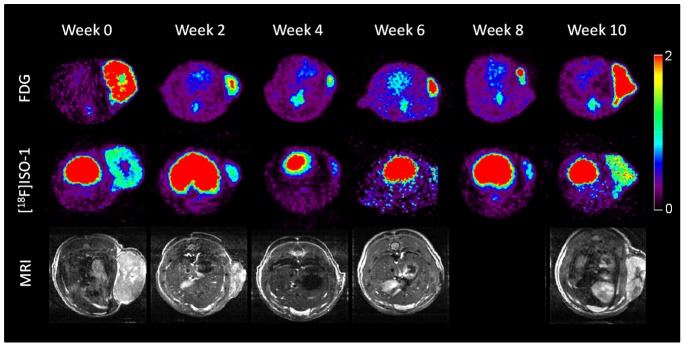
SUV 10-minute sum images at 60-minute post-injection of FDG (top row) and [^18^F]ISO-1 (middle row). Bottom row depicts the corresponding MR image at each time point (week 8 image is missing). Panel depicts treatment of tumor C1-1_L3 with bexarotene. Note that following the imaging session at week 8, treatment was withdrawn.

In bexarotene -treated rats (Figure 6AB), the short-term efficacy (initial 2-weeks) of both FDG and [^18^F]ISO-1 were in agreement with increased tumor volume. Tumor C1-3_R6 did not respond to therapy as characterized by increased volume, FDG uptake, and enhanced ISO-1 signal in the first 2-weeks. Interestingly, in tumor C1-1_L3 both FDG uptake and ISO-1 signal increased between weeks 4–6 while tumor volume remained negligible, as depicted in Figure 7. Removal of bexarotene from the diet at 8 weeks resulted in rapid regrowth of the tumor which was observed in the MRI and PET imaging studies with both FDG and [^18^F]ISO-1.

#### Vorozole-treated rats

Short-term efficacy of Vorozole rats was generally in agreement with changes in MR-derived volume (Figure 6CD). Tumor C2-3_L3 was characterized by increased volume between weeks 0–2 by as much 275% with a concomitant increase in FDG uptake by approximately 140% but with a reduced ISO-1 signal. By week 4, however, tumor volume was 80% of baseline (a reduction in volume by 295% from week 2). Tumor C1-2_L3 exhibited a decrease in volume such that by week 8 the tumor was approximately 2% of the initial volume. Following removal of Vorozole; however, tumor volume increased to 46% from baseline.

## Discussion

FDG PET is an invaluable tool for the clinical diagnosis, staging, and monitoring the response to therapeutic interventions in cancer patients [31]–[33]. In spite of this tremendous clinical success, FDG has its limitations. One potential limitation of FDG is in the imaging of cell proliferation; although tumor cells have a higher metabolic activity than surrounding normal tissue, the uptake of FDG has shown inconsistent results with respect to the correlation of tracer uptake and in vitro measures of cell proliferation [3]. Therefore, there has been a concerted effort in the field to develop alternative strategies for imaging cell proliferation with PET. To date, efforts to image tumor proliferation fall generally into two categories: 1) proliferation rate via imaging the salvage pathway of DNA synthesis using radiolabeled nucleosides which are substrates for TK-1 or TK-2, an enzyme synthesized during the S-phase of the cell cycle in the cytoplasm and mitochondria, respectively. Examples of this approach include [^18^F]FLT [4] and [^18^F]FMAU [34]; and, 2) proliferative status via targeting the sigma-2 receptor. The density of sigma-2 receptors is 10-fold higher in proliferating 66 cells versus quiescent 66 cells both *in vitro* and *in vivo* [15]. This inherent property suggests that the sigma-2 receptor density may provide an imaging biomarker for determining a tumor’s proliferative status, and is likely to be useful in monitoring the antiproliferative activity of potential therapeutic agents.

In order for a new PET radiotracer to be useful in oncological imaging studies it must afford clinically-relevant information that FDG cannot provide. In the first study, we performed breast cancer imaging with PET to evaluate the ability of FDG and [^18^F]ISO-1 to measure the proliferative status, which has been operationally defined as the ratio of proliferating to quiescent tumor cells (P:Q ratio). Previous studies have demonstrated that the density of sigma-2 receptors in membrane homogenates correlated with the P:Q ratio of mouse mammary 66 xenografts [15]. In this work, we correlated the uptake of [^18^F]ISO-1 using small animal PET imaging with flow cytometry measures of the P:Q ratio in this mouse model of breast cancer. As indicated in Figure 1, there was a high correlation between BrdU labeling-derived measures of P:Q and in-vivo measures of [^18^F]ISO-1 uptake. In contrast, there was no correlation between FDG uptake and tumor P:Q ratio. These results are consistent with the report of Haberkorn and colleagues, who found no correlation between FDG uptake and flow-cytometry measures of cell proliferation in a rodent model of breast cancer [35]. Overall, our data indicate that the sigma-2 receptor imaging strategy has a greater potential for predicting the P:Q ratio of a solid tumor than FDG.

In a second study comparing these two radiotracers, the MNU-induced rat mammary carcinoma model was used to evaluate the predictive capacity of FDG and [^18^F]ISO-1 in characterizing tumor growth rate as determined by MRI-derived changes in tumor volume. MNU-induced tumors exhibited characteristic wide range of tumor growth rates as evidenced by the normalized tumor growth profile [22], [36]–[38]. This variability in tumor volume change in MNU-treated rats enabled us to correlate changes in tumor volume with FDG and [^18^F]ISO-1 uptake; the relative percent change in tumor volume between consecutive imaging sessions was used as a measure of the tumors’ proliferation rate. There was a significant correlation between [^18^F]ISO-1 uptake and tumor growth rate as measured by MRI-derived changes in tumor volume. In particular, [^18^F]ISO-1 uptake at a given imaging week significantly correlated with relative change in tumor volume between consecutive imaging sessions. Although there are a number of factors which can influence tumor growth rate, including cell cycle kinetics and birth-death equilibria of tumor cells, the high correlation between [^18^F]ISO-1 uptake and P:Q ratio described above suggests that the rapidly growing MNU-induced tumors have a higher proliferative status than slow growing MNU-induced tumors.

Several recent studies have reported the efficacy of bexarotene and Vorozole on MNU-induced tumors. Of note, recent work by Lubet et al. on the chemopreventive and therapeutic efficacy of bexarotene [22] and Vorozole [38] provide some insight as to the effect of the above mentioned agents. With regard to bexarotene, Lubet and colleagues assessed the preventive effects of bexarotene at 15, 92 and 272 ppm in diet which resulted in tumor preventive effects of 40–100%. Furthermore, a high therapeutic dose of bexarotene 250 ppm caused tumor regression in virtually all animals within 5 weeks. [22]. In the present study we utilized a dose of 250 mg/kg for 8 weeks and observed tumor regression of at least 80% by week 6. In an earlier work, Christov and colleagues [37] examined the therapeutic efficacy of Vorozole and showed that it was highly effective in this ER+ model. In addition to their therapeutic efficiencies, both agents strikingly decreased tumor cell proliferation as assessed by BrdU incorporation within 7 days. Interestingly, studies in a neoadjuvant setting clearly demonstrated that the aromatase inhibitor arimidex, which like vorozole is a high affinity competitive inhibitor, results in profound decreases in Ki67 staining within 2 weeks of treatment in clinical ER+ tumors [39].

The time-course of PET imaging following treatment can be divided into three segments: short-term response to treatment (weeks 0–2), response to withdrawing of treatment at week 8 (weeks 8–10), and the period between weeks 2–8. The first two segments are considered as challenges or stimuli to the tumor. In the first challenge (short-term response), the pattern of FDG uptake and signal due to [^18^F]ISO-1 generally agree with the pattern of tumor growth for both bexarotene and Vorozole. Interestingly, in tumor C2-3_L3 of the Vorozole cohort, decrease in FISO uptake preceded subsequent reduction in tumor volume by 2 weeks, similar to our observations in the untreated group. When looking at PET and MRI time-course patterns most of the tumors treated with Vorozole or bexarotene showed striking regression by MRI within 4 weeks. Interestingly, in humans treated in a neoadjuvant setting, where roughly 50% will show a strong clinical response, it normally requires 4–6 months to observe tumor regression by imaging. In the MNU-induced tumors, profound decreases in tumor cell proliferation were observed within 2 weeks of treatment. In the second challenge (post-withdrawal of therapy), increased uptake of [^18^F]ISO-1 between weeks 8–10 suggested a resurgence of proliferating cells, which was in agreement with the increase in tumor volume. A decrease in [^18^F]ISO-1 uptake may be attributed to either a down-regulation of sigma-2 receptors and/or cell death following treatment. Taken together, further studies are needed to optimize the timing and use of FDG, [^18^F]ISO-1, and other imaging markers following treatment.

In summary, we studied the utility of FDG and [^18^F]ISO-1 in characterizing two different properties of tumors, proliferative status and tumor growth rate. [^18^F]ISO-1 showed a strong correlation between tumor proliferative status whereas no such correlation was observed with FDG. While we did not observe a correlation between FDG uptake and changes in tumor volume, we did observe a significant correlation between [^18^F]ISO-1 uptake and rate of tumor growth rate as defined by changes in tumor volume between consecutive MRI imaging sessions. These data suggest that a given value of [^18^F]ISO-1 uptake provides a predictive measure of an expected change in tumor volume (i.e., growth rate), which attests to the tumor’s aggressiveness. It is expected that such a measure would provide means to optimize a given therapy or combination of therapies based on the kinetic parameters of an individual tumor, thus improving the overall management of cancer.
